# Agreement between cardiac output estimation by multi-beat analysis of arterial blood pressure waveforms and continuous thermodilution in post cardiac surgery intensive care unit patients

**DOI:** 10.1007/s10877-022-00924-z

**Published:** 2022-10-21

**Authors:** Ashish K. Khanna, Lillian Nosow, Lauren Sands, Amit K. Saha, Harshavardhan Agashe, Lynnette Harris, R. Shayn Martin, Bryan Marchant

**Affiliations:** 1 Department of Anesthesiology, Section on Critical Care Medicine, Wake Forest School of Medicine, Atrium Health Wake Forest Baptist Medical Center, Winston-Salem, NC USA; 2 Outcomes Research Consortium, Cleveland, OH USA; 3 Perioperative Outcomes and Informatics Collaborative (POIC), Winston-Salem, NC USA; 4 Wake Forest School of Medicine, Winston-Salem, NC USA; 5 University of Maryland School of Medicine, Baltimore, USA; 6 Department of Anesthesiology, Wake Forest School of Medicine, Atrium Health Wake Forest Baptist Medical Center, Winston-Salem, NC USA; 7 Retia Medical, Valhalla, NY USA; 8 Department of Surgery, Wake Forest School of Medicine, Atrium Health Wake Forest Baptist Medical Center, Winston-Salem, NC 27157 USA; 9 Section on Critical Care Medicine, Section on Cardiac Anesthesiology, Department of Anesthesiology, Wake Forest School of Medicine, Atrium Health Wake Forest Baptist Medical Center, Winston-Salem, NC USA

**Keywords:** Pulse wave analysis, Cardiac output, Hemodynamics, Monitoring, Cardiac surgery, Post-operative, Intensive care unit

## Abstract

We sought to assess agreement of cardiac output estimation between continuous pulmonary artery catheter (PAC) guided thermodilution (CO-CTD) and a novel pulse wave analysis (PWA) method that performs an analysis of multiple beats of the arterial blood pressure waveform (CO-MBA) in post-operative cardiac surgery patients. PAC obtained CO-CTD measurements were compared with CO-MBA measurements from the Argos monitor (Retia Medical; Valhalla, NY, USA), in prospectively enrolled adult cardiac surgical intensive care unit patients. Agreement was assessed via Bland-Altman analysis. Subgroup analysis was performed on data segments identified as arrhythmia, or with low CO (less than 5 L/min). 927 hours of monitoring data from 79 patients was analyzed, of which 26 had arrhythmia. Mean CO-CTD was 5.29 ± 1.14 L/min (bias ± precision), whereas mean CO-MBA was 5.36 ± 1.33 L/min, (4.95 ± 0.80 L/min and 5.04 ± 1.07 L/min in the arrhythmia subgroup). Mean of differences was 0.04 ± 1.04 L/min with an error of 38.2%. In the arrhythmia subgroup, mean of differences was 0.14 ± 0.90 L/min with an error of 35.4%. In the low CO subgroup, mean of differences was 0.26 ± 0.89 L/min with an error of 40.4%. In adult patients after cardiac surgery, including those with low cardiac output and arrhythmia CO-MBA is not interchangeable with the continuous thermodilution method via a PAC, when using a 30% error threshold.

**Supplementary Information:**

The online version contains supplementary material available at 10.1007/s10877-022-00924-z.

## Introduction

Cardiac surgery patients show dynamic and complicated changes in hemodynamics. Initially developed about five decades ago, pulmonary artery catheters (PACs) have long been used for invasive hemodynamic monitoring in these patients [1]. Evidence for mortality, length of stay, cost, survival benefit, infectious morbidity and other complications have been mixed with this intervention [1–5]. Recently, the continuous cardiac output PAC has been shown to pass interchangeability criteria compared to the intermittent thermodilution method, albeit narrowly [6].

Pulse wave analysis devices are a class of minimally-invasive methods which analyze the arterial blood pressure wave form to determine stroke volume and cardiac output [7]. These devices employ algorithms that often model the arterial tree as a two-element Windkessel model. The BP waveform during diastole is modeled as a smoothly falling exponential with a time constant equal to the product of the arterial compliance and resistance [8]. However, a smooth exponential decay during diastole is rarely seen in clinical practice due to distortions caused by wave reflections from the periphery. Multi-Beat Analysis (MBA™) is a novel method that aims to account for the confounding wave reflection. Here multiple beats are used to model the circulation, following which an exponential function is fit to the tail end of the arterial tree impulse response after the distortions due to wave reflection have vanished [8]. In theory, this allows an accurate characterization of systemic vascular resistance and thus cardiac output. The Argos monitor, (Retia Medical; Valhalla, NY, USA) utilizes this proprietary MBA algorithm to allow for beside cardiac output monitoring, and CO is estimated using a 20 s window and updated every 5 s.

Here we prospectively assess the agreement between a continuous PAC guided thermodilution method and cardiac output estimation using the novel MBA method in ICU patients after on-pump cardiac surgery. A previous study had compared the Argos to intermittent thermodilution in 31 patients in the ICU after off-pump cardiac surgery, but that study excluded patients with arrhythmias [9]. Here we expanded the sample size to 79 patients and importantly, assessed agreement in patient with arrhythmias. Continuous CO from the PAC as a reference is uncommon in method comparison studies, nonetheless this method is becoming increasingly common in clinical practice. Being a pragmatic observational study, we used this method as it is the standard of care at our institution.

A cardiac output monitoring method can be considered interchangeable with the intermittent thermodilution method if the difference between the two is less than 30% per the Critchley and Critchley criterion [10]. Peyton and Chong’s meta-analysis comparing minimally invasive methods of CO monitoring to the thermodilution method found an overall error of 41.3% across pulse wave analysis methods including calibrated methods [11]. They made the case that a 45% difference threshold may be more appropriate for minimally invasive methods given that the purpose of these devices is not to replace the PA catheter. In the current observational study, a post-hoc assessment was made in relation to the Critchley and Critchley as well as the Peyton and Chong criteria.

## Methods

### Study design and setting

This study was reviewed and approved by the Wake Forest School of Medicine Institutional Review Board (IRB number and date of approval: Wake Forest School of Medicine (IRB00065503); 6/18/2020). The IRB also determined that this research met the criteria for a waiver of consent entirely according to 45 CFR 46(d). The purpose of this work was to compare CO measurements between the multi-beat analysis method (MBA) and the continuous thermodilution (CTD) method via a pulmonary artery catheter. The study was conducted from July – December 2020 in the cardiovascular surgical intensive care unit of the Wake Forest Baptist Medical Center, a tertiary academic care center part of the Wake Forest School of Medicine.

### Inclusion and exclusion criteria

Adult patients scheduled for elective cardiac surgery (both coronary artery bypass and or valve surgery) on cardiopulmonary bypass, and who received a radial arterial catheter and a pulmonary artery catheter per clinical indications were included in this study. Exclusion criteria included patients less than 18 years of age, those with mechanical circulatory support (e.g., LVADs, ECMO or intra-aortic balloon pumps) or patients with moderate to severe aortic regurgitation, per the contraindications of the Argos monitor.

### Study measurements

After receiving patients in the ICU, standard blood pressure monitoring was continued via the previously established radial arterial catheter and a transducer connected to a bedside multi-parameter monitor (Philips Intellivue, Cambridge, MA). Standard ICU protocols were followed of leveling the pressure transducer to the right atrium and confirming zero of the system to atmospheric pressure. Square wave tests were performed if deemed clinically necessary, at the discretion of the bedside care team. The Argos monitor (Retia Medical LLC., Valhalla, NY, USA) received the radial blood pressure waveform from the bedside monitor via a reusable cable. Patient demographics were entered into the Argos monitor and cable connections were checked to ensure that the blood pressure waveform was being received correctly by the Argos monitor. Subsequently, the front screen of the Argos monitor was covered, so monitoring data was acquired in a user blinded fashion. Research personnel performed intermittent checks of the integrity of the recording system. No patient interventions were performed based on cardiac output numbers on the Argos monitor. Monitoring and data collection continued till patient discharge from the intensive care unit or discontinuation of the arterial line or failure of the arterial line, whichever came first.

Cardiac output readings from the pulmonary artery catheter were calculated continuously by the HemoSphere monitor (Edwards Lifesciences, Irvine, CA, USA) and were recorded once every minute by a connected data capture system (Capsule Medical Device Information Platform, Andover, MA). The Argos monitor records CO-MBA measurements once every 5 s, while the Capsule system records CO-CTD once every minute. After each patient’s ICU stay, data from the Argos monitor and the Capsule system were exported to a computer for further analysis. Data synchronization was ensured by matching the timestamps from the two sources.

### Data processing

100 patients were enrolled, of which 6 were excluded due to unavailability of cardiac output data from the pulmonary artery catheter (CO-CTD). Another 2 subjects were excluded due to removal of the pulmonary artery catheter before application of the Argos monitor. Another 2 subjects were excluded due to blood pressure waveforms unavailable to the Argos monitor (CO-MBA), potentially due to a loose cable connection. One subject was excluded because minute-by-minute CO-CTD could not be extracted from the Capsule system due to technical reasons.

Blood pressure waveforms and continuous thermodilution measurements were visually inspected to determine any artifact or improperly damped waveforms. While the appearance of non-physiological signals is clear, inadequate damping is more difficult to assess. Overdamping, where the blood pressure waveform shows a progressive narrowing of pulse pressure, can be clinically easier to detect and correct, as the cause is often a kink or an air bubble in the transducer tubing [12]. Underdamping results in systolic and diastolic overshoots and causes widening of the pulse pressure and can be more difficult to detect[12]. We inspected the fast flush response when available [13], the waveform morphology, and the maximum systolic slope (dP/dt max) of the BP waveform to determine inadequately damped data, which were excluded from further analysis [14]. As recommended by Romagnoli et al., we used a dP/dt max cutoff of 1.67 mmHg/ms to guide determination of underdamping [14]. Data from 89 subjects was available for further analysis, of which we identified 10 subjects (11%) as having consistently underdamped BP signals and were excluded. In 31 of the remaining 79 subjects, 213 h of data (19%) were partially excluded due to artifact or inadequate damping. After all exclusions, 927 h of data, corresponding to 55,599 CO-CTD and CO-MBA data pairs, were available from 79 subjects. Blood pressure waveform segments were visually inspected by two anesthesiologists blinded to cardiac output measurements, to determine persistent arrhythmia or extrasystoles. These data segments constituted the arrhythmia subgroup. Of these, 4555 data pairs in 26 patients were marked with an arrhythmia or extrasystole label. We also identified data where CO-CTD was less than 5 L/min as the low-CO subgroup and performed analysis of agreement between the two CO measurement methods. CO-CTD was less than 5 L/min in 24,589 data segments from 63 patients.

### Statistical analysis

Continuous thermodilution cardiac output has been shown to have a delayed response, especially under unstable hemodynamic conditions [15]. For the data collected in this study, plotting CO-CTD and CO-MBA onto the same graph revealed that the delay in continuous thermodilution was maximally up to 24 min (supplementary Fig. S1 A, B).

Accordingly, we averaged the continuous thermodilution cardiac output with a one-hour sliding window, while keeping the sampling rate at one measurement every minute, to smooth over any instabilities and delays. CO-MBA was similarly averaged and then resampled to one measurement every minute to allow paired comparisons with CO-CTD. By using an averaging period at least twice as large as the longest delays in the CCO data, we aimed to reduce the influence of short-term changes that could potentially be due to delays in CO-CTD. In this data the time delays in continuous thermodilution were up to 24 min and therefore we used an averaging period of one hour.

Patient demographic distributions were summarized as mean ± standard deviation for continuous quantities (age, height, weight, and BMI) and as integers for the Male/Female distribution. CO-CTD and CO-MBA distributions over all patients were summarized as mean ± standard deviation. CO-CTD and CO-MBA data pairs were analyzed according to correlation and Bland–Altman statistics, considering multiple observations within subjects [16, 17]. Specifically, a linear mixed-effects model was used to model the CO differences between the two methods as the sum of a bias (fixed effect), within-subject variance (random effect) and a between-subject variance (random effect). We calculated the mean and standard deviation of the paired difference between CO-CTD and CO-MBA (bias and precision), the 95% limits of agreement (bias ± 1.96 × precision), and the percentage error [10]. The overall variance used to calculate the precision, limits of agreement and the percentage error was the sum of the within-subject and across-subject variances. All analyses were performed in MATLAB (Mathworks Inc., Natick, USA).

## Results

Patient demographic distributions are summarized in Table 1.

**Table 1:** Baseline patient characteristics. Data are shown as absolute and relative frequencies or mean and standard deviation

Number of included patients, n	79
Age (years)	66 ± 9
Sex, female [n (%)]	21 (27)
Height (cm)	173 ± 10
Weight (kg)	87 ± 23
BMI (kg/m^2^)	29.1 ± 6.6

**Fig. 1:**
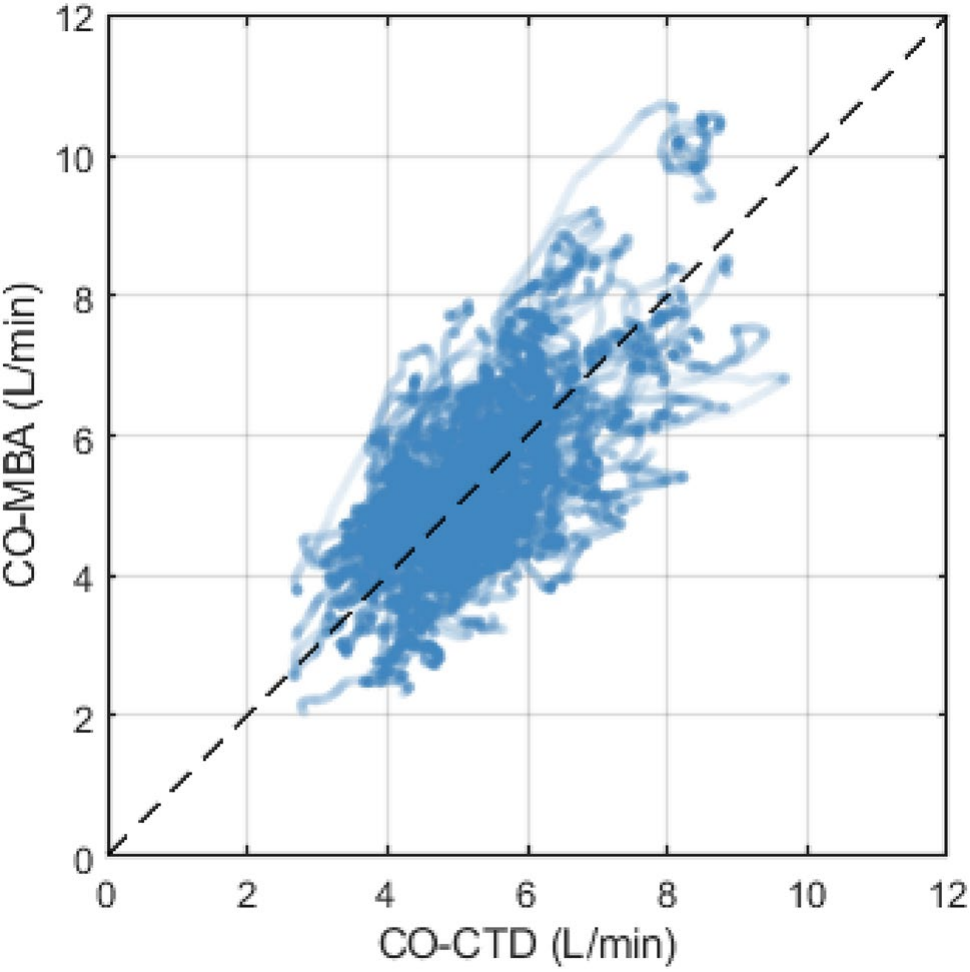
Correlation plot between CO-CTD and CO-MBA

**Fig. 2:**
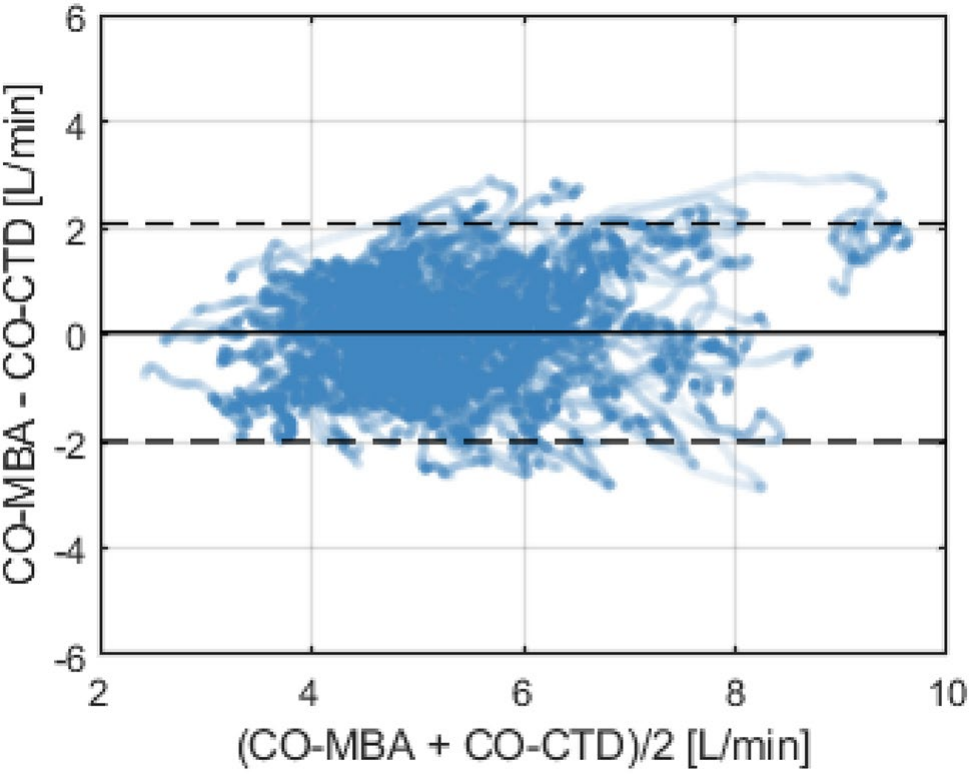
Bland–Altman plot showing agreement between CO-CTD and CO-MBA. Bold horizontal line indicates bias, and dashed lines indicate 95% limits of agreement

Mean value of CO-CTD across all subjects was 5.29 ± 1.14 L/min. Mean CO-MBA was 5.36 ± 1.33 L/min. Correlation between CO-CTD and CO-MBA was 0.64 (Fig. 1). Mean difference between CO-CTD and CO-MBA (bias ± precision) per Bland–Altman analysis was 0.04 ± 1.04 L/min. Limits of agreement were -2.00 to 2.08 L/min (Fig. 2). The percentage error was 38.2%.

**Fig. 3:**
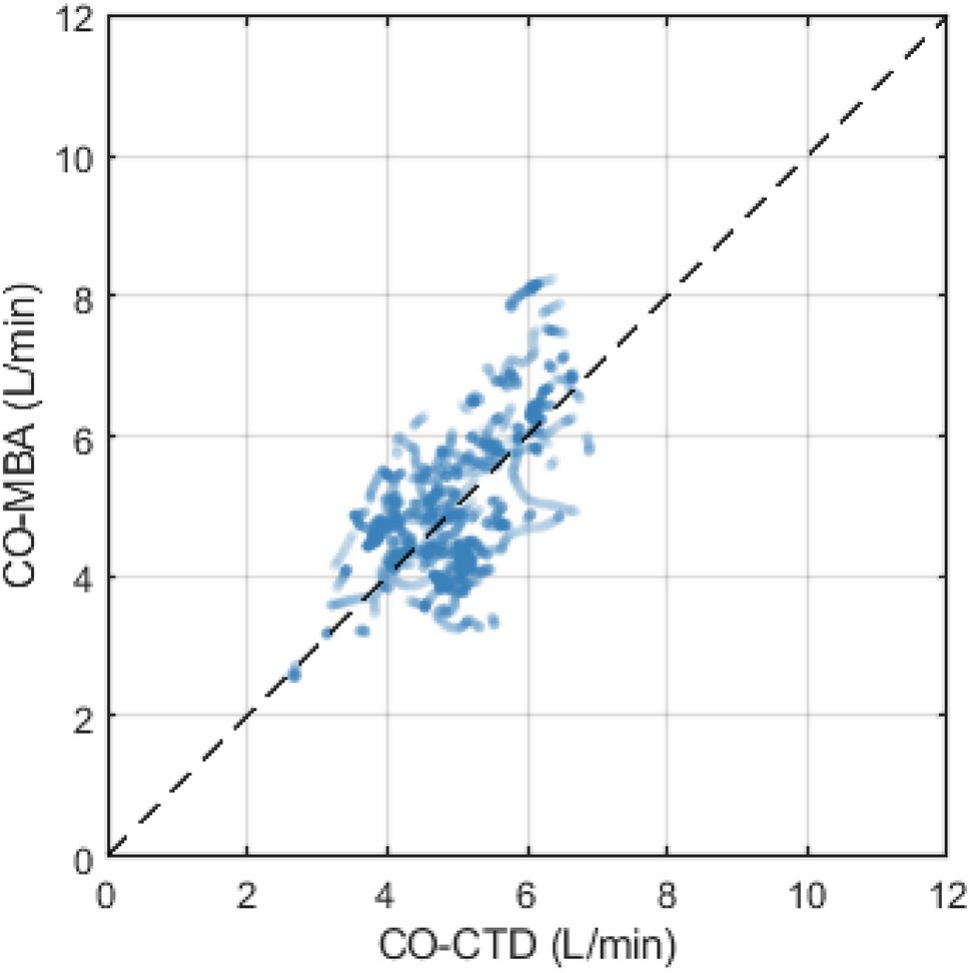
Correlation plot between CO-CTD and CO-MBA, for the arrhythmia subgroup

**Fig. 4:**
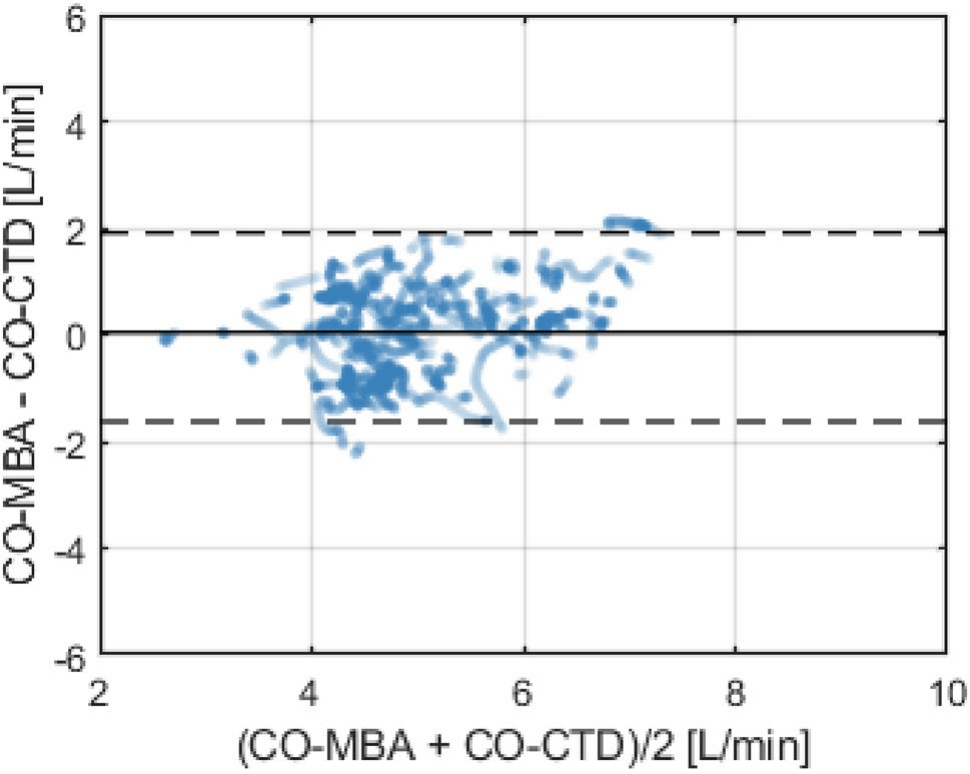
Bland–Altman plot showing agreement between CO-CTD and CO-MBA, for the arrhythmia subgroup. Bold horizontal line indicates bias, and dashed lines indicate 95% limits of agreement

In the arrhythmia subgroup, mean value of CO-CTD across all subjects was 4.95 ± 0.80 L/min. Mean CO-MBA was 5.04 ± 1.07 L/min. Mean difference between CO-CTD and CO-MBA (bias ± precision) per Bland–Altman analysis was 0.14 ± 0.90 L/min. Correlation between CO-CTD and CO-MBA was 0.64 (Fig. 3). Limits of agreement were − 1.63 to 1.91 L/min (Fig. 4). The percentage error was 35.4%. In the low-CO subgroup (CO-CTD < 5 L/min), mean difference between CO-CTD and CO-MBA (bias ± precision) was 0.26 ± 0.89 L/min, with a percentage error of 40.4%.

## Discussion

This prospective analysis of 927 h of data from 79 post-operative cardiac surgery patients recovering in the intensive care unit shows that CO-MBA did not meet the Critchley and Critchley interchangeability criterion with CO-CTD [10]. However, CO-MBA met the Peyton and Chong criteria for clinical acceptance for minimally invasive CO monitoring [11]. This criterion acknowledges that the clinical use of minimally invasive devices is to provide continuous CO monitoring where a PAC is not indicated [18]. Importantly, CO-MBA maintained a similar level of agreement with CO-CTD during arrhythmia and low CO.

Intermittent pulmonary artery thermodilution uses injection of several fluid boluses with a known volume and temperature into the right atrium. The temperature change detected by a distal thermistor is then used to calculate the cardiac output [19]. In contrast, continuous pulmonary artery thermodilution uses a thermal filament in the right ventricle, without the need for manual injections [20]. This method has a variable response time to hemodynamic changes and narrowly passes a 30% error interchangeability criterion with the intermittent bolus thermodilution method and its use as a reference method is a limitation [6]. Nonetheless it is the clinical standard at our institution due to its relative ease of use.

A debate about the merits of PAC placement has been ongoing with multiple large-scale studies both showing and failing to show an overall clinical benefit to placement [1]. Adverse events ranging in severity from self-limiting arrhythmia to pulmonary artery rupture have been reported in the literature with an incidence from two to seventeen percent [2]. Less invasive methods of cardiac output determination, such as the CO-MBA, may allow the intensivist to supplement a PAC in situations where extended periods of monitoring may be necessary. Where a PAC is not strongly indicated due to some of the limitations discussed above, minimally invasive methods such as CO-MBA provide an alternative to monitor CO.

We identified 10 subjects (11%) as having consistently underdamped blood pressure signals on the arterial line and were excluded. For comparison, other investigators have previously excluded 9 out of 40 subjects (23%) and found 92 out of 300 subjects (31%) to have underdamping or resonant arterial lines in similar patient populations [9, 14]. The percentage of subjects excluded due to improper damping in this study is lower, possibly due to missing square wave tests, and is a limitation. We used recommended objective criteria to guide the determination of improper damping of BP waveforms as much as possible, however there is a subjective component that could have affected the data and is a limitation of the study.

Up to a third of patients have atrial and ventricular arrhythmias after cardiac surgery [21]. Some of these rhythm changes last for a significant amount of time and are associated with hemodynamic instability. This may result in periods of under perfusion and trigger organ system injury. We specifically analyzed a subgroup of patients with rhythm disturbances identified by two anesthesiologists and found agreement between the two methods to be consistent with the overall agreement. This consistency of cardiac output measurements during rhythm instability shown in this work is of critical significance. Even though we only had 26 patients in this subgroup, our work serves as hypothesis generating for future analysis where specific types, times and durations of rhythm changes could be analyzed in larger cohorts.

Greiwe and colleagues previously compared CO-MBA to intermittent thermodilution in the post-cardiac surgery intensive care unit [9]. They had a smaller sample size than our report (167 comparison points; 31 patients) and performed bolus thermodilution cardiac output measurements at seven pre-defined time points. Furthermore, this was not a real time data collection with a bedside monitor, rather was an offline analysis where arterial blood pressure waveforms were fed into the Argos monitor retrospectively. Percentage error reported was 40.7% and not very different from the 35–40% range in our cohorts. Our results are also consistent with the CO-MBA technique method comparison analysis reported in the cardiac operating room by Saugel and colleagues [22]. CO estimations showed reasonable agreement and trending ability between the two methods, with a concordance rate of 89%. Another recent study comparing the Argos and FloTrac monitors, showed that Argos had a higher concordance rate with intermittent thermodilution and may prove valuable in CO trending [23]. However, both devices were not interchangeable with thermodilution for absolute CO measurement due to high percentage errors of 50%. In non-cardiac surgery, the MBA method was 93% concordant with the transesophageal Doppler method during both fluid and vasopressor administration [24].

There are some limitations to this analysis. While we allowed for adjustments for CO response time differences between the two comparison methods by averaging both methods over a one-hour time scale, this may have the effect of reducing the variance in both methods. The choice of one hour as the averaging window is subjective, and may influence results. We are unsure of the clinical validity and relevance of an earlier detection of a change in cardiac output, however, our delay in response time reported (12–24 min) for the continuous thermodilution PAC is not different from published work reporting a response time of 10 min or more [15]. Furthermore, the arrythmia subgroup involved a small sample size, was of uncertain clinical significance including the actual hemodynamic changes involved, and the specific nature or duration of the rhythm disturbances. This was novel all the same, and relevant since rhythm disturbances are very common in the post-operative cardiac surgery patient. BP waveform quality was checked in a large majority of our patients, per clinical standard of care at the discretion of the bedside caregiver. While this is a limitation since the waveform quality should be checked with a fast flush/square wave test before every comparison, it does represent actual clinical practice rather than a research setting. While removing artifactual, underdamped or overdamped arterial line waveforms and data, we could have introduced a selection bias. Such periods of less-than-optimal monitoring constitute a non-trivial part of the total monitoring time of a critically ill patient in the ICU. In CO method comparison studies with intermittent thermodilution as the reference method, a four-quadrant concordance analysis is suggested to assess trending agreement. Here, because the reference method was continuous thermodilution with consecutive samples being one minute apart, a meaningful four-quadrant analysis could not be performed. In addition, we did not plan for comparisons at specific interventions such fluid loading or rapid changes in inotropy or afterload and could not perform a trending analysis. Finally, this work comes from a single center and may be reflective of local practice patterns precluding generalizability.

## Conclusions

Cardiac output calculated using a multi-beat analysis is not interchangeable with continuous thermodilution CO in adult patients recovering in the intensive care unit after on-pump coronary artery bypass surgery. In arrhythmia and low CO subgroups, the multi-beat analysis method remained outside the interchangeability criterion of 30%.

## Supplementary Information

Below is the link to the electronic supplementary material.

Supplementary file1 (DOCX 236 kb)

## Abbreviations

(ICU): Intensive care unit
(BMI): Body mass index
(AKI): Acute kidney injury
(KDIGO): Kidney Disease: Improving Global Outcomes
