# Supplementary material for: Agreement between cardiac output estimation by multi-beat analysis of arterial blood pressure waveforms and continuous thermodilution in post cardiac surgery intensive care unit patients
Source: J Clin Monit Comput. 2022 Oct 21;37(2):559–65. doi: 10.1007/s10877-022-00924-z (PMC10068656; doi:10.1007/s10877-022-00924-z)
Supplement: Supplementary file 1 — Supplementary file1 (DOCX 236 kb) [file 10877_2022_924_MOESM1_ESM.docx]

**Supplementary figures:** Examples comparing the time delay in CO-CTD to CO-MBA are shown in Fig. S1. The time delay can be variable (Fig. S1(A) and S1(B)). If this time delay is not considered, the differences between the two methods can be artificially inflated. For e.g., in Fig S1(A), comparing CO-MBA to CO-CTD at 2315 hrs. leads to CO-MBA = 3.5 L/min and CO-CTD = 5.1 L/min, with a difference of 1.6 L/min between the two. Considering a time delay of 20 minutes in CO-CTD, the comparison should be made at 2335 hrs., when CO-CTD = 3.6 L/min and the difference between the two methods is 0.1 L/min. Accounting for delays in this manner is not always feasible as the delays in CO-CTD are variable and the magnitude of the time delay is not always clear. We used a 1-hr moving average over both signals (CO-CTD and CO-MBA) to reduce the influence of these artificial errors due to the time delay.


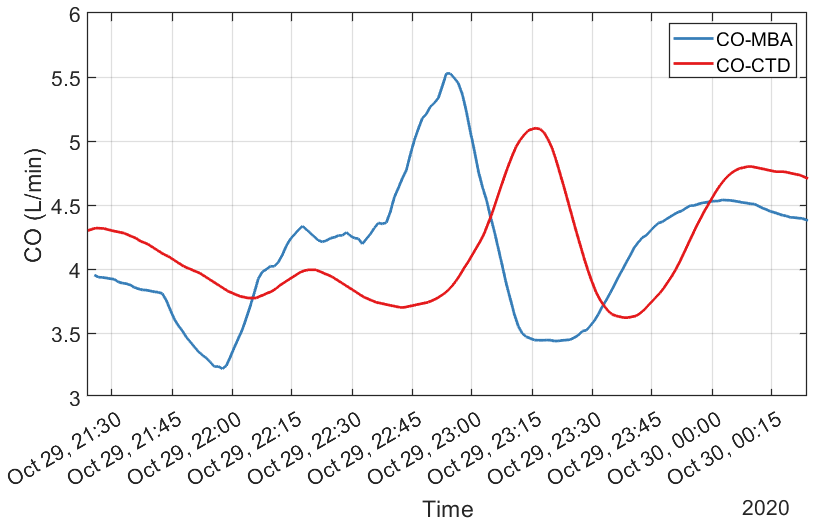

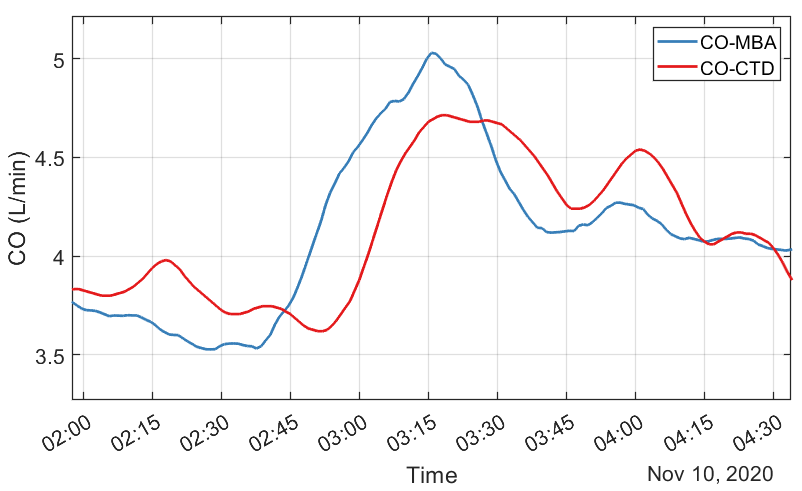


**A**

**B**

**Fig S1.** **CO-CTD and CO-MBA show similar but time-shifted patterns of CO changes. In the top plot (A), the time delay for CO-CTD appears to be around 20 minutes, while for another subject in the bottom plot (B) the delay appears to be 15 minutes.**
